# Behavior Change Techniques and Their Mechanisms of Action: A Synthesis of Links Described in Published Intervention Literature

**DOI:** 10.1093/abm/kay078

**Published:** 2018-10-10

**Authors:** Rachel N Carey, Lauren E Connell, Marie Johnston, Alexander J Rothman, Marijn de Bruin, Michael P Kelly, Susan Michie

**Affiliations:** 1 Centre for Behaviour Change, University College London, 1-19 Torrington Place, London WC1E 7HB, UK; 2 Department of Kinesiology, University of Rhode Island, 25 W Independence Way, Kingston, RI 02881, USA; 3 Aberdeen Health Psychology Group, Institute of Applied Health Sciences, University of Aberdeen, Aberdeen, UK; 4 Department of Psychology, University of Minnesota, Minneapolis, MN, USA; 5 Primary Care Unit, Institute of Public Health, University of Cambridge, Cambridge, UK

**Keywords:** Behavior change, Theory, Methodology, Behavior change technique, Mechanism of action, Evidence synthesis

## Abstract

**Background:**

Despite advances in behavioral science, there is no widely shared understanding of the “mechanisms of action” (MoAs) through which individual behavior change techniques (BCTs) have their effects. Cumulative progress in the development, evaluation, and synthesis of behavioral interventions could be improved by identifying the MoAs through which BCTs are believed to bring about change.

**Purpose:**

This study aimed to identify the links between BCTs and MoAs described by authors of a corpus of published literature.

**Methods:**

Hypothesized links between BCTs and MoAs were extracted by two coders from 277 behavior change intervention articles. Binomial tests were conducted to provide an indication of the relative frequency of each link.

**Results:**

Of 77 BCTs coded, 70 were linked to at least one MoA. Of 26 MoAs, all but one were linked to at least one BCT. We identified 2,636 BCT–MoA links in total (mean number of links per article = 9.56, *SD =* 13.80). The most frequently linked MoAs were “Beliefs about Capabilities” and “Intention.” Binomial test results identified up to five MoAs linked to each of the BCTs (*M* = 1.71, range: 1–5) and up to eight BCTs for each of the MoAs (*M =* 3.63, range: 1–8).

**Conclusions:**

The BCT–MoA links described by intervention authors and identified in this extensive review present intervention developers and reviewers with a first level of systematically collated evidence. These findings provide a resource for the development of theory-based interventions, and for theoretical understanding of intervention evaluations. The extent to which these links are empirically supported requires systematic investigation.

## Teaser

This study produced a large dataset of hypothesised links between behaviour change techniques and the processes through which they change behaviour, as described in a set of published research reports. These findings can be used in the design of behaviour change interventions, and in furthering our understanding of how interventions have their effects.

Behavior plays a key role in maintaining health, and in the prevention, management, and treatment of disease and disability. Activities such as smoking, alcohol misuse, physical inactivity, and certain dietary behaviors contribute to the global disease burden and often lead to premature death [1, 2]. There has been a steady global increase in diseases attributed to behavioral risk factors, with substantial associated losses in national income. The need for effective and cost-effective health-related behavior change interventions is acute.

Despite rapid growth in behavioral intervention research, the effects of behavioral interventions continue to be typically small, variable, and not maintained long-term [3, 4]. Cumulative progress in the design of more effective interventions could be improved by developing a more widely shared understanding of the “mechanisms of action” (MoAs) through which interventions bring about change [5]. A more thorough understanding of how and why interventions achieve their effects, through identification of the links between behavior change techniques (BCTs) and the MoAs they target, would enable us to (i) design interventions that include components more likely to be effective [6] and (ii) better explain intervention effects.

Behavioral interventions are often delivered as part of complex systems that include a number of BCTs. A BCT is defined as a replicable component of an intervention designed to alter or redirect causal processes that regulate behavior (i.e., a technique is proposed to be a potentially “active ingredient”). BCTs are designed to enable behavior change, and can do this by augmenting factors that facilitate behavior change, or by mitigating factors that inhibit behavior change. As an example, one might hypothesize that the BCT “Graded Tasks” (defined as: “set easy-to-perform tasks, making them increasingly difficult, but achievable, until behavior is performed” [7]) might change behavior by *increasing* beliefs about one’s capabilities. On the other hand, one might hypothesize that the BCT “Restructuring the Social Environment” (defined as: “change, or advise to change, the social environment in order to facilitate performance of the wanted behavior or create barriers to the unwanted behavior” [7]) might change behavior by *decreasing* negative social influences.

BCTs are usually selected on the basis of the theoretical constructs they are proposed to target; for example, prompting experience of mastery through behavioral practice is often used to increase self-efficacy, based on Bandura’s theory of self-efficacy [8]. However, links between the full range of BCTs that exist and the theoretical constructs they are believed to modify are not clearly understood, and the rationale underlying BCT selection is not always transparent in intervention articles.

To enhance the design of more effective interventions, we need to develop a clearer understanding of the processes through which individual BCTs have their effects (i.e., their MoAs) [9]. We conceptualize these MoAs as a range of theoretical constructs that represent the processes through which a BCT affects behavior. In this context, MoAs are constructs specified in theories of behavior and behavior change that can be seen to “mediate” intervention effects, such as “beliefs about capabilities,” “knowledge,” and “behavioral regulation.” They can be characteristics of the individual (i.e., intrapersonal psychological processes) and characteristics of the social and physical environment (e.g., social support).

Understanding the links between BCTs and MoAs is important not just for intervention development (i.e., for the purpose of selecting appropriate BCTs), but also for evaluation (i.e., for understanding the processes through which BCTs have their effects). Intervention evaluations, replications, and syntheses have benefitted, in recent years, from the development of guidance for reporting interventions, such as the Template for Intervention Description and Replication (TIDieR) [10], and BCT Taxonomy version 1 (BCTTv1) [7, 11]. BCTTv1 is a classification system for characterizing the potentially active ingredients of behavioral interventions. It has been used to specify intervention techniques across a wide range of behavioral domains, for example physical activity [12], alcohol use [13], medication adherence [14], condom use [11], and behavior of health professionals [15]. It has also been applied in evidence syntheses to retrospectively identify BCTs used in published interventions and to evaluate their efficacy [16–19].

Such frameworks and taxonomies are helpful methods for knowledge accumulation and evidence synthesis; however, despite these advances, we currently lack a thorough understanding of the links between BCTs and specific MoAs. To advance understanding of these links, one approach is to review the published intervention literature and identify the links that have been identified by their authors. Previous research has suggested that empirical evidence about the links between individual BCTs and their MoAs may be limited [20]. However, by examining links that are explicitly described or hypothesized by authors within published articles of behavior change interventions [21], we can provide a first level of systematically collated evidence to shed light on the rationale researchers provide underlying their BCT selection, and help to elucidate the assumptions made by researchers about how intervention strategies have their effects.

This article reports on the first study from a larger program of research [9], examining links between BCTs and their MoAs. The current study aimed to identify the frequency with which specific BCTs are described as linked to specific MoAs. We drew on the published intervention literature to draw out the often-implicit assumptions made by researchers about (i) how to target theoretical constructs of interest (i.e., which BCTs target specific MoAs) and (ii) how interventions work (i.e., through which MoAs specific BCTs influence behavior). We also sought to understand whether or not any BCT–MoA links appeared with a relatively high level of frequency across the intervention literature.

As part of this program of research [9], the following specific research questions were addressed in this study (i.e., according to the published intervention literature):

1. How frequently is each possible BCT–MoA link described?
2. Which BCTs are frequently described as targeting a specific MoA?
3. Which MoAs are frequently described as influenced by a specific BCT?
4. Do any specific BCT–MoA links occur more frequently than might be expected given the average frequency of BCT–MoA links?

## Methods

### Procedure

We identified published articles reporting behavior change interventions (both development and evaluation) in which authors described links between BCT(s) and MoA(s) (although they were not necessarily explicitly labeled as a “behavior change technique” or “mechanism of action” by the authors). To maximize efficiency, given time and resource constraints, our search strategy prioritized articles in which (i) BCTs had been identified using a taxonomy (BCTTv1, or one of the previous cross-behavior, or behavior-specific, taxonomies described in the introduction), either in the article itself (by intervention authors), or retrospectively by systematic reviewers, and/or (ii) MoAs had been identified using a theoretical framework. We identified articles through electronic searches, requests to experts, and by reviewing the reference lists of systematic reviews.

#### Electronic searches

To identify articles in which BCTs were likely to have been explicitly identified (to maximize efficiency of data extraction), we conducted a forward-search (i.e., a search of citations of a given paper) of five published BCT taxonomies [7, 22–25]. To identify articles in which MoAs were likely to have been explicitly specified, we conducted a forward-search of the Theory Coding Scheme [26] and Theoretical Domains Framework [27, 28]. All forward-searches were conducted within two online databases: Web of Science and Google Scholar.

#### Requests to experts

We sent a request for relevant articles to the 42 members of the project’s International Advisory Board (http://www.ucl.ac.uk/behaviour-change-techniques/people/iab), spanning 10 countries, and to researchers in the field via scientific and professional societies, including the US Society for Behavioral Medicine (SBM), European Health Psychology Society (EHPS), UK Society for Behavioural Medicine, and Division of Health Psychology of the British Psychological Society.

#### Reference lists of systematic reviews

The reference lists of all systematic reviews identified through the search methods above, including a review published by NICE as part of its behavior change guidance [29], were reviewed. Relevant articles were downloaded and screened for inclusion. By including intervention articles in which BCTs and/or MoAs had been coded retrospectively (i.e., through systematic review coding), we were not restricted to intervention articles that used the language of our set of BCTs and/or MoAs (e.g., articles in which a BCT was described using different labels to those used in the BCT Taxonomy). This also meant that we were not restricted to intervention articles that were dated after the publication of the various framework papers above.

#### Inclusion criteria

Intervention articles were included if they provided the description or evaluation of a behavior change intervention, and if the author(s) explicitly described a BCT (not necessarily labeled as such by the authors) as linked to one or more MoA(s) (i.e., there had to be at least one explicit, identifiable link between a BCT and an MoA). For example, an article would be included if the authors described an intervention that asked participants to set goals related to the target behavior, and indicated that goal-setting would change behavior through increasing self-regulation. Articles were excluded if they were not peer-reviewed (e.g., unpublished doctoral theses), if no behavioral outcome was reported, and/or if descriptions were not sufficiently detailed to be able to identify at least one link. For example, an article would be excluded if the authors described the intervention in detail, including BCTs, but did not explicitly state *how* any of the BCTs were expected to change the target behavior. Articles were also excluded where multiple BCTs were linked to multiple MoAs, but the specific links described were unclear. For example, an article would be excluded if it contained a table with a list of BCTs and a list of MoAs, where it was not possible to tell whether or not the authors were proposing that all of the BCTs were linked to all of the MoAs. No restrictions were made for year of publication, target behavior, journal, study quality, or article type.

### Data Extraction

#### Screening

Two researchers initially reviewed the full texts of all identified articles for eligibility, with screening guidelines iteratively updated and all discrepancies resolved through discussion. Once acceptable inter-rater reliability was achieved (Kappa = 0.9), articles were screened independently (see Supplementary File 1 for a summary of inter-rater reliability across all stages of screening and coding).

#### BCT coding

BCTs were extracted from the included intervention articles using BCTTv1 [7, 11] according to guidelines adapted from those on the BCTTv1 online training website (www.bct-taxonomy.com; see Supplementary File 2 for BCT coding guidelines). Examples of guidelines for BCT coding included: that BCTs should only be coded if they targeted one or more of the target behaviors or key preparatory behaviors of the intervention, that the whole intervention description should be read before beginning to code BCTs, and that, where BCTs were previously coded in the intervention articles using BCTTv1, the authors’ original coding was maintained; where an earlier taxonomy had been used [22], coding was updated in line with BCTTv1 guidelines. Two researchers who were trained in BCT coding independently coded BCTs (regardless of whether or not they were linked to an MoA) until inter-rater reliability was acceptable (Prevalence and Bias Adjusted Kappa [PABAK] = 0.9; see Supplementary File 1), at which point articles were coded initially by one researcher, and subsequently checked by one of two other researchers. Inter-rater reliability for BCT coding was assessed using PABAK [30], which accounts for high prevalence of negative agreement [11].

#### Coding links between BCTs and MoAs

Following BCT coding, links between BCTs and MoAs were extracted from the articles by two researchers independently. Coding these links was an iterative process, where discrepancies were resolved through discussion and coding guidelines revised accordingly (see Supplementary File 2). As we did not use a “finite” number of MoAs in data extraction, we used percentage agreement, rather than Kappa, to calculate reliability between coders.

A theoretical construct was extracted as an MoA provided it was (i) defined as a process through which behavior change could occur and (ii) clearly linked to a BCT. Discrepancies were resolved through discussion, and consulting with senior experts (S.M., M.J., A.J.R., M.d.B., M.P.K.) where needed. Guidelines for BCT–MoA link coding were revised when judged necessary to improve clarity (see Supplementary File 2 for final set of coding guidelines). Examples of BCT–MoA link coding guidelines included: that each BCT–MoA link should only be extracted once in any intervention description and that the most specific links possible should be coded (e.g., if BCT X was linked to “reinforcing factors” as an MoA, and reinforcing factors was said to include “feedback mechanisms and peer support,” BCT X was linked to feedback mechanisms and peer support, rather than “reinforcing factors”). To guide our coding, we drew on a set of 26 general MoAs; these were the 14 domains from the Theoretical Domains Framework [27] and the 12 additional most frequent MoA constructs from a set of 83 theories of behavior change [31] (see Appendix F of Supplementary File 2 for a full list of these 26 MoAs).

### Data Synthesis

Extracted data were tabulated as follows. General information about the study (e.g., author, year, article and study type, target behavior, whether the authors identified a theoretical model as underpinning the development of the intervention) was entered into a “source” table; all identified BCTs were recorded in a “BCT” table; BCT–MoA link data were extracted into a “link” table. In the link table, each BCT–MoA link was assigned a unique row, to which the following information was added by two coders: BCT identity number (from BCTTv1 taxonomy), MoA label and definition (as described by the intervention authors), explicitness of the link (1 = *some inference needed* and 2 = *no inference needed*), whether or not the links included *groups* of BCTs or MoAs (1 = *one BCT linked to one MoA* and 2 = *more than one BCT linked to one MoA or more than one MoA linked to one BCT*), and whether the link was tested empirically (1 = *MoA not measured and BCT–MoA link not tested*, 2 = *MoA measured but BCT–MoA link not tested*, and 3 = *BCT–MoA link tested*). The three tables were connected using an identifying code to ensure all data were available for each article.

Following data extraction, authors’ definitions of MoAs were categorized into the set of 26 general MoAs described earlier (i.e., 14 domains from the TDF [27] and 12 frequent MoA constructs from 83 theories of behavior change [31]). Two coders categorized MoAs until inter-coder reliability was >90% (see Supplementary File 3 for guidelines). Discrepancies were resolved through discussion, and MoAs that could not be categorized into any of the 26 were categorized as “other.”

### Analysis

To address our first three research questions (i.e., how frequently each possible BCT–MoA link is described, which BCTs are frequently described as targeting a specific MoA, which MoAs are frequently described as influenced by a specific BCT), we conducted descriptive analyses (in MS Excel) to examine the frequency of links between BCTs and MoAs (i.e., the number of articles in which a particular link was described).

In addition, to examine the relative frequency of each BCT–MoA link (i.e., our fourth research question), a series of one-tailed exact binomial tests was conducted (using *R* statistical software [32]) on the links for which MoAs could be categorized, comparing the observed with the expected frequency of occurrence for each link. In the absence of an agreed expected frequency of BCT–MoA links that could be used for comparison (i.e., H_0_), we computed an expected value to serve as an estimate of the frequency that might be observed if BCTs were randomly linked to MoAs. The expected value was computed as the probability that a particular BCT was coded (frequency with which the BCT was linked with any MoA / total number of links between all BCTs and MoAs) multiplied by the probability a particular MoA was coded (frequency with which the MoA was linked with any BCT / total number of links between all BCTs and MoAs).

The resulting *p* values represent an indication of the likelihood of a link, allowing us to examine how frequently a specific BCT (X) was linked to a specific MoA (Y), by comparing this frequency with how often BCT X was used in *any* intervention, and how often MoA Y was targeted in *any* intervention. Because of the method used to compute an expected value, the resulting comparisons serve to identify links that are high in frequency *relative* to other links examined in this set of studies. Thus, a particular link may emerge as relatively frequent despite being identified a small number of times, *if* the BCT and/or MoA was rarely identified across the interventions. For example, if BCT X was linked with MoA Y only twice, but it was the *only* time MoA Y was linked to any BCT, the likelihood of this link may be greater than would be expected by chance (and would therefore be high in *relative* frequency, with a low *p* value). Conversely, if BCT X was linked to MoA Y eight times, but BCT X and MoA Y were both frequently linked to a range of other MoAs/BCTs, the likelihood of this link may *not* be greater than would be expected by chance (and would therefore be lower in relative frequency, with a larger *p* value).

We used *p* < .05 as an arbitrary minimum criterion for a BCT–MoA link, although clearly more or less stringent criteria can be applied to the resulting data. We are not making statistical inferences about links that meet, or do not meet, this criterion; rather, we are presenting the data in this way as an indication of the relative frequency of each link. We used a one-tailed test as the aim was to identify agreed-upon links rather than their absence.

### Heat Maps

The full results of the analyses (i.e., not just those that did not reach this threshold) are represented in “heat maps” of the findings. Heat maps allow individual data values to be represented as colors within a matrix to aid in interpreting the findings, and were generated through *R* [32]. The cells within the heat map contain a numerical value (i.e., *p* values) and are colored or shaded to reflect the relative strength or “heat” of that value (in this case, the relative frequency of a particular link).

The heat map clusters rows (i.e., BCTs) and columns (i.e., MoAs) by similarity, such that BCTs linked to similar MoAs are clustered together, and MoAs linked to similar numbers of BCTs are clustered together. MoAs that could not be categorized into one of our 26 (e.g., where there was not enough information in the article or the definition was unique and did not map on to any of our 26 definitions) were not included in these binomial tests. Thus, although we have selected one criterion for what constitutes a “link,” all of the data are available such that others can select alternative criteria as needed.

## Results

### Characteristics of Included Studies

Of 974 intervention articles retrieved, 697 (72%) were excluded based on full-text screening. The most common reasons for exclusion were that intervention articles specified constructs as MoAs, but there were no clear links to individual BCTs, or that intervention articles linked BCTs to theoretical constructs, but did not identify these constructs as MoAs or hypothesized mediators. Of the remaining articles, 277 described at least one link, with years of publication ranging from 1982 to 2016 and 49% published in or after 2010. More than 10 behaviors were targeted by the interventions, including physical activity (40%), dietary behaviors (18%), alcohol reduction (10%), and smoking (6%). A majority (78%) were articles reporting outcome evaluations (rather than development papers or protocols). Approximately 14% of the articles did not mention any theoretical basis for the intervention. Thirteen percent of the articles mentioned theory, but without specifying how theory was applied to intervention development or evaluation. The analyses and discussions that follow are based on the 277 included articles. A full summary of study characteristics can be found on Open Science Framework (OSF) at https://osf.io/7qjvn/.

### Characteristics of Extracted Links

A total of 2,636 BCT–MoA links were extracted from the 277 articles, of which 33% required some inference to code, and 0.9% had been empirically tested within the included study. There were approximately 10 links per study (*M* = 9.56, *SD =* 13.80), of which 88% included a *group* of BCTs linked to one MoA, or a group of MoAs linked to one BCT; 12% included a single BCT and a single MoA. Seventy-seven BCTs (of the 93 in BCTTv1) were coded across the 277 articles, 70 of which were linked to at least one MoA. The BCTs that were most frequently linked to an MoA were “Instruction on How to Perform the Behavior” (182 times) and “Problem Solving” (177 times). The most frequently linked MoA was “Beliefs about Capabilities” (734 times), followed by “Intention” (318 times). One of the MoAs from our pre-existing set of 26 was not identified: “Norms” (defined as “the attitudes held and behaviors exhibited by other people within a social group”). A full list of the 2,636 BCT–MoA links is available at https://osf.io/7qjvn/.

### Do Any Specific BCT–MoA Links Occur More Frequently Than Might Be Expected Given the Average Frequency of BCT–MoA Links?

Binomial tests were conducted to examine the relative frequency of BCT–MoA links. There were 87 links that met the criterion of *p* < .05, including 51 of 93 (55%) BCTs and 24 of 26 (92%) MoAs.

Up to eight BCTs were identified for each of the MoAs (*M =* 3.63, range: 1–8), and up to five MoAs were identified for each of the BCTs (*M* = 1.71, range: 1–5). For example, the MoA “Social Learning/Imitation” was linked to one BCT: “Demonstration of Behavior,” whereas the MoA “Attitude Towards the Behavior” was linked to eight BCTs: “Information about Health Consequences,” “Salience of Consequences,” “Information about Social and Environmental Consequences,” “Information about Emotional Consequences,” “Pros and Cons,” “Material Incentive (Behavior),” “Framing/Reframing,” and “Incompatible Beliefs.” Similarly, for BCTs, “Information about Health Consequences” was linked to the MoAs: “Knowledge,” “Beliefs about Consequences,” “Intention,” “Attitude Towards the Behavior,” and “Perceived Susceptibility/Vulnerability.”

Of the 25 MoAs (from our set of 26) that were linked to a BCT at least once, only “Optimism”—derived from the Theoretical Domains Framework [27]—was not linked to any BCT at the *p* < .05 threshold. Several BCTs, on the other hand, were coded frequently but did not meet the *p* < .05 threshold for any MoA. For example, the BCT “Review Behavior Goals” was coded 36 times, and “Social Support (Emotional)” was coded 14 times, but the relative frequency with which these were linked to an MoA did not meet the *p* < .05 threshold.

**Fig. 1.**
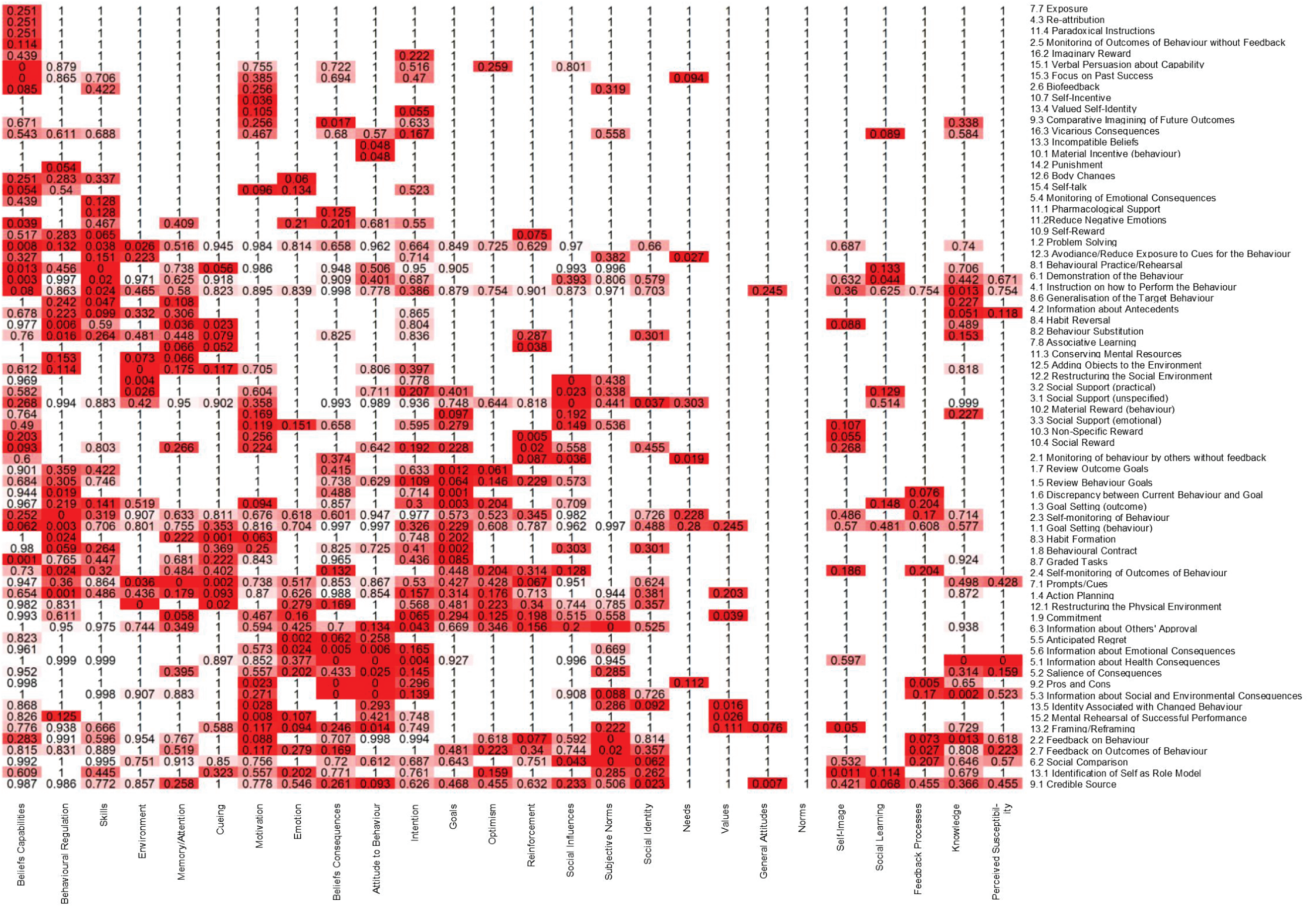
Heat map representing the relative frequency of BCT–MoA links. Each cell contains a numerical value (i.e., *p* value) and is colored to reflect the relative “heat” of that value (in this case, the relative frequency of a particular link). Knowledge = an awareness of the existence of something; Skills = an ability or proficiency acquired through practice; Social/ Professional Role and Identity = a coherent set of behaviors and displayed personal qualities of an individual in a social or work setting; Beliefs about Capabilities = beliefs about one’s ability to successfully carry out a behavior; Optimism = confidence that things will happen for the best or that desired goals will be attained; Beliefs about Consequences = beliefs about the consequences of a behavior (i.e., perceptions about what will be achieved and/or lost by undertaking a behavior, as well as the probability that a behavior will lead to a specific outcome); Reinforcement = processes by which the frequency or probability of a response is increased through a dependent relationship or contingency with a stimulus or circumstance; Intention = a conscious decision to perform a behavior or a resolve to act in a certain way; Goals = mental representations of outcomes or end states that an individual wants to achieve; Memory, Attention and Decision Processes = ability to retain information, focus on aspects of the environment, and choose between two or more alternatives; Environmental Context and Resources = aspects of a person’s situation or environment that discourage or encourage the behavior; Social Influences = those interpersonal processes that can cause oneself to change one’s thoughts, feelings or behaviors; Emotion = a complex reaction pattern involving experiential, behavioral, and physiological elements; Behavioral Regulation = behavioral, cognitive, and/or emotional skills for managing or changing behavior; Norms = the attitudes held and behaviors exhibited by other people within a social group; Subjective Norms = one’s *perceptions* of what most other people within a social group believe and do; Attitude towards the behavior = the general evaluations of the behavior on a scale ranging from negative to positive; Motivation = processes relating to the impetus that gives purpose or direction to behavior and operates at a conscious or unconscious level; Self-Image = one’s conception and evaluation of oneself, including psychological and physical characteristics, qualities, and skills; Needs = deficit of something required for survival, well-being, or personal fulfillment; Values = moral, social, or aesthetic principles accepted by an individual or society as a guide to what is good, desirable, or important; Feedback Processes = processes through which current behavior is compared against a particular standard; Social Learning/Imitation = a process by which thoughts, feelings, and motivational states observed in others are internalized and replicated without the need for conscious awareness; Behavioral Cueing = processes by which behavior is triggered from either the external environment, the performance of another behavior, or from ideas appearing in consciousness; General Attitudes/Beliefs = evaluations of an object, person, group, issue, or concept on a scale ranging from negative to positive; Perceived Susceptibility/Vulnerability = perceptions of the likelihood that one is vulnerable to a threat. *BCT* behavior change technique; MoA mechanism of action.

A heat map visually representing the frequency of BCT–MoA links (with darker colors representing *p* values closer to zero) is shown in Fig. 1. These data are also available online as part of an interactive online tool (https://theoryandtechniquetool.humanbehaviourchange.org/; see Discussion section for more details).

Table 1 describes the 51 BCTs and the MoAs to which they were most frequently linked. Thus, this table provides a summary of the MoAs through which these 51 BCTs may affect behavior, according to the authors of this set of published interventions. In some cases, there is one clear MoA for a given BCT; for example, the BCTs Goal Setting (Behavior) and Action Planning—both frequently coded across interventions—were only linked to “Behavioral Regulation” (*p* = .003 and *p* =.001, respectively). In other cases, there are BCTs with links to multiple MoAs, but with one seemingly “dominant” MoA; for example, while the BCT “Problem Solving” was frequently linked to three MoAs, the link to “Beliefs about Capabilities” (*p* = .008; occurring 65 times) was substantially more frequent than the next highest two: “Environmental Context and Resources” (*p* = .026; occurring 9 times) and “Skills” (*p* = .038; occurring 18 times).

**Table 1:** BCT–MoA links with relatively high frequency in 277 intervention articles: Organized by BCT and presented in order of (i) BCT frequency from this study and (ii) *p* values

BCT	MoA	Frequency	*p* Value
Instruction on how to perform the behavior (4.1)	Knowledge Skills	17 20	.013 .024
Goal setting (behavior) (1.1)	Behavioral regulation	15	.003
Problem solving (1.2)	Beliefs about capabilities	65	.008
	Environmental context and resources	9	.026
	Skills	18	.038
Social support (unspecified) (3.1)	Social influences	34	<.001
	Social/professional role and identity	5	.037
Demonstration of the behavior (6.1)	Beliefs about capabilities skills	60 17	.003 .020
	Social learning/imitation	3	.044
Action planning (1.4)	Behavioral regulation	14	.001
Feedback on behavior (2.2)	Subjective norms	19	<.001
	Knowledge	13	.013
Information about health consequences (5.1)	Knowledge Beliefs about consequences	18 26	<.001 <.001
	Attitude towards the behavior	19	<.001
	Perceived susceptibility/vulnerability	10	<.001
	Intention	28	.004
Behavioral practice/rehearsal (8.1)	Skills Beliefs about capabilities	24 47	<.001 .013
Social comparison (6.2)	Subjective norms	31	<.001
	Social influences	9	.043
Information about social and environmental consequences (5.3)	Beliefs about consequences Attitude towards the behavior	20 16	<.001 <.001
	Knowledge	13	.002
Self-monitoring of behavior (2.3)	Behavioral regulation	18	<.001
Credible source (9.1)	General attitudes/beliefs	2	.007
	Social/professional role and identity	4	.023
Adding objects to the environment (12.5)	Environmental context/resources	8	<.001
Prompts/cues (7.1)	Memory, attention, and decision processes	8	<.001
	Behavioral cueing	6	.002
	Environmental context/resources	5	.036
Graded tasks (8.7)	Beliefs about capabilities	28	<.001
Pros and cons (9.2)	Beliefs about consequences	12	<.001
	Attitude towards the behavior	9	<.001
	Feedback processes	3	.005
	Motivation	5	.023
Framing/reframing (13.2)	Self-image	2	<.050
	Attitude towards the behavior	7	.014
Behavior substitution (8.2)	Behavioral regulation	5	.016
Social reward (10.4)	Reinforcement	3	.020
Focus on past success (15.3)	Beliefs about capabilities	23	<.001
Restructuring the physical environment (12.1)	Environmental context/resources	9	<.001
	Behavioral cueing	3	.020
Behavioral contract (1.8)	Goals	4	.002
Information about other’s approval (6.3)	Subjective norms Intention	13 12	<.001 .043
Verbal persuasion about capability (15.1)	Beliefs about capabilities	27	<.001
Feedback on outcomes of behavior (2.7)	Subjective norms Feedback processes	5 2	.020 .027
Reduce negative emotions (11.2)	Beliefs about capabilities	12	.039
Salience of consequences (5.2)	Attitude towards the behavior	4	.025
Commitment (1.9)	Values	1	.039
Self-monitoring of outcomes of behavior (2.4)	Behavioral regulation	5	.024
Information about emotional consequences (5.6)	Beliefs about consequences Attitude towards the behavior	6 5	.005 .006
	Emotion	2	.024
Goal setting (outcome) (1.3)	Goals	4	.003
Social support (practical) (3.2)	Social influences	4	.023
	Environmental context and resources	3	.026
Discrepancy between current behavior and goal (1.6)	Goals Behavioral regulation	3 3	.001 .019
Avoidance/reducing exposure to cues for the behavior (12.3)	Needs	1	.027
Identification of self as role model (13.1)	Self-image	2	.011
Restructuring the social environment (12.2)	Environmental context/resources Social influences	3 6	.004 <.001
Nonspecific reward (10.3)	Reinforcement	2	.005
Habit formation (8.3)	Behavioral cueing	3	.001
	Behavioral regulation	3	.024
Review outcome goals (1.7)	Goals	2	.012
Mental rehearsal of successful performance (15.2)	Motivation Values	3 1	.008 .026
Material incentive (behavior) (10.1)	Attitude towards the behavior	1	.048
Monitoring of behavior by others without feedback (2.1)	Needs Social influences	1 2	.019 .036
Generalization of target behavior (8.6)	Skills	2	.047
Comparative imagining of future outcomes (9.3)	Beliefs about consequences	3	.017
Identity associated with changed behavior (13.5)	Values Motivation	1 2	.016 .028
Anticipated regret (5.5)	Emotion	2	.002
Habit reversal (8.4)	Behavioral regulation	4	.006
	Behavioral cueing	2	.023
	Memory, attention, and decision processes	2	.036
Associative learning (7.8)	Reinforcement	1	.038
Self-incentive (10.7)	Motivation	1	.036
Incompatible beliefs (13.3)	Attitude towards the behavior	1	.048

Numbers in parentheses for each BCT are as per BCTTv1. *BCT* behavior change technique; MoA mechanism of action; *BCTTv1* BCT Taxonomy version 1.

It is also clear, based on the data in Table 1, that the links may reflect hypothesized causal pathways, rather than mutually exclusive targeted constructs; for example, the BCT “Information about Health Consequences” was linked to the MoAs “Knowledge” (*p* < .001), “Beliefs about Consequences” (*p* < .001), “Attitude Towards the Behavior” (*p* < .001), “Perceived Susceptibility/Vulnerability” (*p* < .001), and “Intention” (*p* = .004).

Table 2 describes the 24 MoAs and the BCTs to which they were most frequently linked. This table therefore provides a summary of the BCTs that could potentially be used to target these 24 MoAs, according to the authors of this set of published interventions. Again, in some cases, there is one clear BCT for a given MoA; for example, the MoA “Perceived Susceptibility/Vulnerability” was only linked to “Information about Health Consequences” (*p* < .001), and the MoA “Social Learning/Imitation” was only linked to “Demonstration of the Behavior” (*p* = .044). In other cases, multiple (theoretically linked) BCTs were linked to a given MoA; for example, the MoA “Emotion” was linked to “Anticipated Regret” (*p* = .002), and “Information about Emotional Consequences” (*p* = .024).

**Table 2:** BCT–MoA links with relatively high frequency in 277 intervention articles: Organized by MoA alphabetically and presented in order of *p* values.

MoA	BCT	Frequency	*p* Value
Attitude towards the behavior	Information about health consequences (5.1)	19	<.001
	Information about social and environmental consequences (5.3)	16	<.001
	Pros and cons (9.2)	9	<.001
	Information about emotional consequences (5.6)	5	.006
	Framing/reframing (13.2)	7	.014
	Salience of consequences (5.2)	4	.025
	Material incentive (behavior) (10.1)	1	.048
	Incompatible beliefs (13.3)	1	.048
Behavioral cueing	Habit formation (8.3)	3	.001
	Prompts/cues (7.1)	6	.002
	Restructuring the physical environment (12.1)	3	.020
	Habit reversal (8.4)	2	.023
Behavioral regulation	Self-monitoring of behavior (2.3)	18	<.001
	Action planning (1.4)	14	.001
	Goal setting (behavior) (1.1)	15	.003
	Habit reversal (8.4)	4	.006
	Behavior substitution (8.2)	5	.016
	Discrepancy between current behavior and goal (1.6)	3	.019
	Self-monitoring of outcomes of behavior (2.4)	5	.024
	Habit formation (8.3)	3	.024
Beliefs about capabilities	Graded tasks (8.7)	28	<.001
	Verbal persuasion about capability (15.1)	27	<.001
	Focus on past success (15.3)	23	<.001
	Demonstration of the behavior (6.1)	60	.003
	Problem solving (1.2)	65	.008
	Behavioral practice/rehearsal (8.1)	47	.013
	Reduce negative emotions (11.2)	12	.039
Beliefs about consequences	Information about health consequences (5.1)	26	<.001
	Information about social and environmental consequences (5.3)	20	<.001
	Pros and cons (9.2)	12	<.001
	Information about emotional consequences (5.6)	6	.005
	Comparative imagining of future outcomes (9.3)	3	.017
Environmental context and resources	Restructuring the physical environment (12.1)	9	<.001
	Adding objects to the environment (12.5)	8	<.001
	Restructuring the social environment (12.2)	3	.004
	Problem solving (1.2)	9	.026
	Social support (practical) (3.2)	3	.026
	Prompts & cues (7.1)	5	.036
Emotion	Anticipated regret (5.5)	2	.002
	Information about emotional consequences (5.6)	2	.024
Feedback processes	Pros and cons (9.2)	3	.005
	Feedback on outcomes of behavior (2.7)	2	.027
General attitudes/beliefs	Credible source (9.1)	2	.007
Goals	Discrepancy between current behavior and goal (1.6)	3	.001
	Behavioral contract (1.8)	4	.002
	Goal setting (outcome) (1.3)	4	.003
	Review outcome goals (1.7)	2	.012
Intention	Information about health consequences (5.1)	28	.004
	Information about others’ approval (6.3)	12	.043
Knowledge	Information about health consequences (5.1)	18	<.001
	Information about social and environmental consequences (5.3)	13	.002
	Instruction on how to perform the behavior (4.1)	17	.013
	Feedback on behavior (2.2)	13	.013
Memory, attention, and decision processes	Prompts/cues (7.1)	8	<.001
	Habit reversal (8.4)	2	.036
Motivation	Mental rehearsal of successful performance (15.2)	3	.008
	Pros and cons (9.2)	5	.023
	Identity associated with changed behavior (13.5)	2	.028
	Self-incentive (10.7)	1	.036
Perceived susceptibility/ vulnerability	Information about health consequences (5.1)	10	<.001
Needs	Monitoring of behavior by others without feedback (2.1)	1	.019
	Avoidance/reducing exposure to cues for the behavior (12.3)	1	.027
Reinforcement	Nonspecific reward (10.3)	2	.005
	Social reward (10.4)	3	.020
	Associative learning (7.8)	1	.038
Self-image	Framing/reframing (13.2)	2	<.050
	Identification of self as role model (13.1)	2	.011
Skills	Behavioral practice/rehearsal (8.1)	24	<.001
	Demonstration of the behavior (6.1)	17	.020
	Instruction on how to perform the behavior (4.1)	20	.024
	Problem solving (1.2)	18	.038
	Generalization of target behavior (8.6)	2	.047
Social influences	Social support (unspecified) (3.1)	34	<.001
	Restructuring the social environment (12.2)	6	<.001
	Social support (practical) (3.2)	4	.023
	Monitoring of behavior by others without feedback (2.1)	2	.036
	Social comparison (6.2)	9	.043
Subjective norms	Feedback on behavior (2.2)	19	<.001
	Social comparison (6.2)	31	<.001
	Information about other’s approval (6.3)	13	<.001
	Feedback on outcomes of behavior (2.7)	5	.020
Social learning/imitation	Demonstration of the behavior (6.1)	3	.044
Social/professional role and identity	Credible source (9.1)	4	.023
	Social support (unspecified) (3.1)	5	.037
Values	Identity associated with changed behavior (13.5)	1	.016
	Mental rehearsal of successful performance (15.2)	1	.026
	Commitment (1.9)	1	.039

Numbers in parentheses for each BCT are as per BCTTv1. *BCT* behavior change technique; MoA mechanism of action; *BCTTv1* BCT Taxonomy version 1.

## Discussion

Findings from this study represent the first dataset summarizing hypothesized links between BCTs and MoAs that were frequently described by authors of published interventions. We identified 2,636 BCT–MoA links between 70 BCTs and 25 MoAs. Of those, 87 links met the *p* < .05 criterion. Identifying these links provides an initial resource of theoretical and practical value indicating which links are believed to be present (i.e., BCT X is frequently linked with MoA Y) and which links appear to be absent (i.e., BCT X is frequently identified but never linked with MoA Y).

In some cases, there is one clear BCT for a given MoA and one clear MoA for a given BCT. In other cases, there are BCTs linked to more than one MoA and MoAs linked to more than one BCT. There are a number of possible explanations for this—for example, for some constructs, relevant BCTs have been explicitly described in the theoretical literature. Intervention strategies to target self-efficacy (a conceptually identical construct to “Beliefs about Capabilities,” as defined in this study), for instance, have been explicitly identified in Bandura’s theory of self-efficacy [8]; the definitions Bandura provides of mastery experience, vicarious experience, and verbal persuasion are similar to the BCTs “Behavioral Practice/Rehearsal,” “Demonstration of the Behavior,” and “Verbal Persuasion about Capability,” respectively, all of which were linked to “Beliefs about Capabilities” in this study. In our study, “Beliefs about Capabilities” was the most frequently identified MoA across all articles, which may reflect the relative clarity with which this construct has been linked to BCTs within behavioral theories.

There are also BCTs and MoAs for which no clear links emerged. For example, there were two MoAs from our set of 26 for which no links were identified at the *p* < .05 level: “Optimism” (confidence that things will happen for the best or that desired goals will be attained) and “Norms” (the attitudes held and behaviors exhibited by other people within a social group). One possible explanation for this is that there may be a lack of clarity or agreement in the behavioral science community regarding the BCTs that can be used to target these MoAs. This is particularly problematic given that the MoA “Norms” occurs frequently in behavioral theories (see [31]). An alternative explanation is that researchers do not see these MoAs as being “modifiable” by BCTs, but rather see them as representing aspects of the individual (e.g., dispositional optimism) or environment (e.g., cultural norms) that may be difficult or impossible to target in behavioral interventions.

This research has also highlighted BCT–MoA links that have been identified but are infrequently used. For example, the BCT “Problem Solving” was linked to the MoA “Behavioral Regulation” 13 times; this did not meet the *p* < .05 criterion.

The heat map, and Tables 1 and 2, can be viewed as a summary of intervention researchers’ beliefs about BCT–MoA links, and can be used as a starting point for intervention designers and evaluators. These data can be drawn upon to identify BCTs that have the potential to target relevant MoAs (e.g., for the purpose of intervention development) and, conversely, to understand the MoAs that individual BCTs are designed to target (e.g., for the purpose of intervention evaluation and theory development). To identify the likely “optimal” BCT–MoA link(s) (e.g., for the purpose of planning an intervention), one can refer to Tables 1 and 2, which list the links that met the *p* < .05 criterion.

For instance, a researcher interested in increasing perceived vulnerability/susceptibility (e.g., drawing on the Extended Parallel Process Model [33]) may consider, based on our findings, that an appropriate BCT might be to provide information about the health consequences of the depicted unsafe/unhealthy behavior. Although some of the frequently identified links are intuitive, there are others that may be less immediately obvious (e.g., the link between the BCT “Mental Rehearsal of Successful Performance” and the MoA “Values”). By drawing on these findings, researchers may identify creative ways in which to target MoAs of interest (e.g., by including less commonly used BCTs).

Our findings can also be used to develop a framework for designing and conducting empirical tests of the BCT–MoA links, to guide the development of an evidence base that can resolve ambivalence about links, and to explore the potential of BCTs and MoAs that appear to be currently underused. Thus, the BCT–MoA links database can be used both to identify links that have been frequently described in the literature, for which empirical tests are needed, as well as to identify links that appear to be understudied. More broadly, advancing the science of behavior change at a theoretical and methodological level, through this and similar initiatives (e.g., see www.scienceofbehaviourchange.org), helps to provide the grounding on which researchers and practitioners can build innovative interventions (e.g., by combining BCTs, knowing where important gaps are, and providing a basis for new hypotheses).

There are a number of additional points to emerge from this research. Seventy-two percent of the articles identified through our search methods did not explicitly describe links between BCTs and MoAs. These findings are consistent with previous meta-analytic findings, which indicated that, although 50% of the interventions reviewed reported a theoretical basis, 90% did not report links between all BCTs and individual theoretical constructs [20]. A common thread among guidelines for intervention development and evaluation [29, 34] is the need for a strong and rigorously applied theoretical basis to optimize effectiveness and enhance our understanding of intervention effects [35]. Although many interventions state that they draw on theory when developing interventions, when descriptions of the links between theoretical constructs and individual BCTs are lacking, it can be difficult to draw generalizable theoretical conclusions.

Our findings point to a more general issue relating to theory use that has hampered intervention research: that conceptualizations of what constitutes “theory-based” are highly variable. A large number of interventions that are reported to be based on theory in fact draw on implicit or partially applied theories [20, 36, 37]. It is often unclear whether and/or how theory has been used in the selection of BCTs, and in the targeting and measurement of theoretical constructs that are considered to be mediating variables in the change process. Simply describing an intervention as having been informed by theory does not mean it *has* been [5]. To maximize the potential usefulness of theory, it is crucial that intervention articles replace implicit assumptions about how interventions have their effects with explicit statements as to how and why theoretical principles guiding the design of the intervention were applied and tested [31, 38–40].

Finally, the finding that a majority of BCTs and MoAs were not linked individually by authors, but instead as groups of BCTs or MoAs may indicate that authors considered that there were synergistic relationships among BCTs and/or among MoAs (e.g., BCTs A, B, and C and/or MoAs X, Y, and Z work together in the behavior change process). Alternatively, it could point to a lack of specificity in the selection of BCTs and the targeting of MoAs, and/or to a lack of detail in intervention reporting.

### Limitations

A number of limitations of the current work should be noted. The studies in this review were purposively selected to maximize the likelihood of identifying BCT–MoA links. In our call for articles, we contacted international societies with broad reach in North America (SBM) and Europe (EHPS); however, there are international societies covering other parts of the world that may have elicited further international research articles. Our dataset of articles may not be representative of the wider behavioral intervention literature; for example, the intervention literature may be restricted in the theories represented, and/or in how they represent the theories. We would note, however, that we did not set out to conduct a systematic review or to identify a representative sample of intervention articles; our aim was to identify a corpus of literature in which BCT–MoA links were most likely to be identifiable.

It should also be noted that the links extracted from the 277 articles were based on authors’ descriptions, and very few had been tested empirically within the articles. This suggests a clear research agenda: for researchers in behavior change to systematically test the links that have been frequently described. To this end, an initiative is underway in the United States to advance efforts to identify, measure, and manipulate MoAs through the experimental medicine approach (www.scienceofbehaviourchange.org; see also [41]).

The results of this study provide no information about the links that did not appear in the included study articles. The absence of reported links may reflect several possibilities: authors’ beliefs that such links do not exist, not considering these links when designing their studies, finding them too difficult to operationalize, using theoretical constructs and ideas implicitly, defaulting to common-sense assumptions about how behavior change happens, or not including this detail when reporting.

Finally, there are other characteristics of the studies, and BCT–MoA links, that we did not extract and that may be of interest and relevance (e.g., type of behavior change, such as initiation or maintenance, hypothesized interactive effects among BCTs, etc.). By publishing our dataset online through OSF and through our interactive online tool (see below), we welcome further data extraction and/or additional analyses by researchers who are interested in examining these characteristics. The findings from this study are informing a subsequent study that is bringing together other characteristics such as BCT delivery, behavioral target, intervention setting, and target population (see www.humanbehaviourchange.org).

### Future Research Directions

This is one of three related studies examining the links between BCTs and (i) MoAs and (ii) behavioral theories [9]. Findings from the current study will be triangulated with those from an expert consensus study involving 105 behavior change experts [42]. The triangulation exercise will provide an additional body of evidence by comparing the results in this study with current thinking by experts in the field, which will address many of the limitations associated with literature-based evidence. Together, these two data sets will provide an integrated matrix that will draw together the links described in published articles with those agreed by experts in the field. This suite of studies forms a key part of a larger program of research building an “ontology” of behavior change interventions that will extend relationships to modes of delivery, exposure, types of behaviors, populations, settings, and intervention effects (see www.humanbehaviourchange.org). This program of work aims to advance our methods for intervention design, evaluation, and synthesis, creating an up-to-date knowledge base that can be tailored to specific populations, settings, and target behaviors.

### Data Sharing

The data sets resulting from this study are available via the website OSF (https://osf.io/7qjvn/) to ensure they are maximally transparent and useful to the scientific community. Publishing the data on OSF will enable research groups to identify new research questions and share data. By publishing our full matrix of links with all relevant data, we encourage researchers to examine the full matrix of links when drawing on the findings.

The findings from this study have contributed to two online resources for the research community. First, the BCT coding completed as part of this work (i.e., for 277 articles) is available as part of an existing resource that collates interventions specified by BCTs (see www.bct-taxonomy.com/interventions). This online resource is searchable by author, year, BCT, and target behavior, and also includes a facility for researchers to add articles that have identified BCTs using BCTTv1. Second, the “heat map” matrix is available as an interactive tool for researchers and intervention developers (https://theoryandtechniquetool.humanbehaviourchange.org/). For each link, users are able to access current study data, upload other data and information about relevant research activities, and contribute suggestions for collaborative research efforts to populate the matrix with empirical evidence (www.humanbehaviourchange.org). The more that programs of research in this area can be co-ordinated, the more efficiently evidence about BCT–MoA links will accumulate.

## Supplementary Material

kay078_suppl_Supplementary_File_1

kay078_suppl_Supplementary_File_2

kay078_suppl_Supplementary_File_3
