# Supplementary material for: Behavior Change Techniques and Their Mechanisms of Action: A Synthesis of Links Described in Published Intervention Literature
Source: Ann Behav Med. 2018 Oct 10;53(8):693–707. doi: 10.1093/abm/kay078 (PMC6636886; doi:10.1093/abm/kay078)
Supplement: kay078_suppl_Supplementary_File_1 [file kay078_suppl_supplementary_file_1.pdf]

Supplementary File 1: Inter-coder reliability for intervention report coding

Stage of coding	Index	Reliability	# Papers coded before reliability achieved
Screening	Kappa	0.9	22
BCT Coding	PABAK	0.9	93
Link Coding	Percentage Agreement	90%	183

Note: Since there was not a 'finite' number of possible MoAs, we used percentage agreement – and not Kappa – for links coding.
