# Supplementary material for: Behavior Change Techniques and Their Mechanisms of Action: A Synthesis of Links Described in Published Intervention Literature
Source: Ann Behav Med. 2018 Oct 10;53(8):693–707. doi: 10.1093/abm/kay078 (PMC6636886; doi:10.1093/abm/kay078)
Supplement: kay078_suppl_Supplementary_File_2 [file kay078_suppl_supplementary_file_2.pdf]

Supplementary File 2: Coding Guidelines

BCT Coding: Guidelines

1. Definition of a BCT**:** A replicable component of an intervention designed to alter or redirect causal processes that regulate behaviour; that is, a technique is proposed to be an ‘active ingredient’ (e.g. feedback, self-monitoring, and reinforcement).
   i. BCTs contain verbs (*e.g., provide, advise, arrange*) that refer to the action(s) taken by the person/s delivering the technique. BCTs can be delivered by an 'interventionist' or self-delivered.
   ii. BCTs contain the term "behaviour" referring to a single action or sequence of actions that includes the performance of wanted behaviour(s) and/or inhibition (non-performance) of unwanted behaviour(s).
2. Coders should familiarise themselves with BCTs (e.g., labels, definitions and examples), and should read and re-read BCT definitions as many times as needed (i.e. to have a good understanding of what must be specified in the intervention description in order for the BCT to be coded). The whole intervention description should be read before beginning to code BCTs. There may be multiple BCTs within one sentence.
3. Assess and rate how certain you are of the identification of the BCT, noting whether you think the BCT is (i) present in all probability (with a “+” symbol) or (ii) present beyond all reasonable doubt (“++”) – mark this in your comment on PDF.
4. BCTs are relevant to behaviours at both i**ndividual** and **group** levels. For example, the following are both examples of the same BCT, but the first is at an individual level while the second is at a group level.
   i. “Individual medical practitioners compared their number of patient referrals with the number made by their colleagues” [BCT: Social comparison ++]
   ii. “The number of referrals made by the health practice was compared with that of other health practices locally” [BCT: Social comparison ++]
5. All BCTs within intervention descriptions should be coded and added to the BCT coding mastersheet - even those not linked to a mechanism of action.
6. Where BCTs have not already been coded within papers, the most recent BCT taxonomy of 93 BCTs, BCTTv1 (Michie et al., 2013), should be used. Where BCTs have been coded using an earlier version of the taxonomy, these should be re-coded using BCTTv1 to standardise coding. BCTs that are the same across taxonomies will not be recoded, provided coders agree with the previous coding. If the intervention was previously coded by a review, the coding should be added to the intervention papers (if not already available).
7. BCTs should only be coded if they are targeting one or more of the target behaviours or key preparatory behaviours of the intervention - not supportive behaviours that are active ingredients in *engaging* the person. Thus, while we would include ‘condom buying’ in a safe sex intervention, we would not include ‘parent/child’ communication in a dietary intervention (where the behaviour measured is fruit & veg intake). This is due to (a) the complexity of this process, (b) the time constraints of the project and (c) the lack of clarity afforded to causal sequences within intervention descriptions. If unsure whether ‘preparatory’ behaviour or not, keep aside for further review.
8. If the intervention description includes a label from the BCT taxonomy (e.g. ‘problem solving’), but the description of this BCT appears to contradict the definition from the BCT taxonomy (e.g. ‘problem solving activities that asked participants to decide if statements were true or false’), do not code.
9. For the purposes of this project, motivational interviewing (MI), counselling, stress management and other composite techniques, *when not broken down further*, should **not** be coded as BCTs.

BCT-MoA Links Coding: Guidelines

1) Definition of a Mechanism of Action: The process through which a BCT affects behaviour.
2) For each BCT, examine for presence of link to a mechanism of action (see Table 1 below). In order to be coded as a link, the author must hypothesise that BCT(s) X changes behaviour through Mechanism(s) Y, where Mechanism Y is specified as a mechanism of action (i.e. there must be an explicit link to behaviour).

Table 1: Coding BCT-mechanism of action links

**Code as Mechanism of Action if:**	**Do not code as Mechanism of Action if:**
- It is labelled a 'determinant' or 'mediator' of behaviour<br>- It is explicitly hypothesised to change behaviour (wording includes: 'influences', 'has an effect on'/'affects', 'changes')	- Authors have only described the mechanism of action in relation to previous research (and it is not considered to be a mechanism of action in the context of the current study)<br>- It is unclear whether a construct is an mechanism of action or a BCT<br>- A construct is specified as a mechanism of action, but the measurement of the construct is a measure of the degree of implementation of, or engagement with, a BCT, rather than a mediator between the BCT and behaviour (see Figure 1 below). For example, if action planning is said to have an influence on behaviour and is measured by asking participants whether or not they set an action plan.<br>- A theory is stated as a theory of behaviour change, and the construct in question is termed a 'key construct' of that theory, but not specified to be a 'key construct' for behaviour change.
**Code as Link if:**	**Do not code as link if:**
- One BCT has been explicitly hypothesised to link to one or more mechanisms of action, or one mechanism of action has been explicitly hypothesised to link to one or more BCTs.<br><br>**Example:**<br>- 'We hypothesised that attitude towards the behaviour would mediate the effect of the normative feedback (containing BCTs X, Y & Z) on behaviour'.	- Two or more BCTs are linked to 2 or more mechanisms of action, and there are no clear 1-1 or n-1 links.<br>o If authors clearly state 2 BCTs work through both of 2 MoAs, ok to include (e.g. authors state that BCT X and BCT Y both influence behaviour through both of MoA A and MoA B).<br>o Note 5a coding guideline.<br><br>**Example:**<br>- Table with groups of BCTs linked to groups of mechanisms of action.<br>- 'Social norms, attitude and self-efficacy should mediate effect of the social comparison and social support components'

3) If the intervention evaluation paper refers to a previously published protocol paper, check this protocol paper for any extra links (i.e. links that are not in the evaluation paper). Code all BCTs and links in the evaluation paper in the first instance, and code any extra links in the protocol paper. If there are unlinked BCTs in secondary paper, do not code these extra BCTs.
4) Where there is a sequence of mechanisms of action targeted (i.e. BCT-attitude-intention-behaviour), code all direct and indirect links. 'Explicitness' should be coded as 1 where links are indirect.
5) If a BCT is linked to multiple mechanisms of action, which are branched into one theoretical construct, code the most specific links possible. For example, if BCT X is linked to 'reinforcing factors', and reinforcing factors is said to contain 'feedback mechanisms and peer support', code BCT X as linked to (i) feedback mechanisms and (ii) peer support, and not 'reinforcing factors'.
   a) If more than one BCT is linked to an overarching theoretical construct (e.g. reinforcing factors) that is branched into multiple mechanisms, you can code all links. E.g. BCT X, and BCT Y linked to (i) feedback mechanisms, and (ii) peer support.
6) Each BCT-mechanism of action link should only be extracted once in any intervention description (i.e. the only time a BCT from the same intervention appears in the links spreadsheet more than once is if it is linked to different mechanisms).
7) Tailoring: If a construct is clearly specified as a mechanism of action, and an intervention delivers specific BCTs to target participants with certain values of the mechanism of action, those BCTs can be linked to the mechanism of action. For example, if an intervention specifies self-efficacy as a mechanism of action, and participants with low self-efficacy are given specific BCTs, these BCTs can be linked to self-efficacy.
8) BCTs that are also specified as mechanisms of action: The behaviour change process follows a sequence (as pictured in Figure 1 below). Some intervention papers specify mediating effects which are the extent to which a BCT was implemented, or engaged with by intervention participants. While these effects do mediate the effect of an intervention as planned on behaviour change, these effects are considered part of the fidelity of the intervention process, and not the mechanisms through which interventions have their effect (see Bellg, 2004 *Health Psychology* for more reference; new paper also addresses this: Lippke et al., 2016).

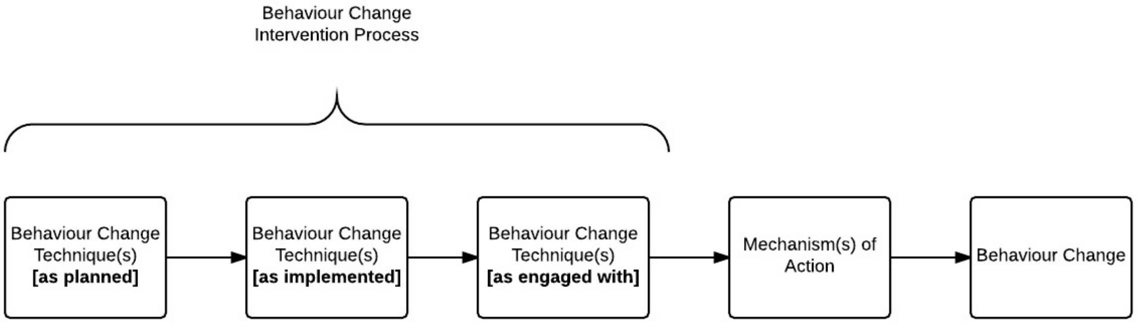

Figure 1: Behaviour change sequence

APPENDIX A:
Data Extraction for Source Table

**Field**	**Data**
Source ID	Code denoting source ID (first 4 letters of first author's name + year, e.g. MICH13)
Researcher (initials)	Initials of researcher extracting data
Author	Name of first author
Year	Year of publication
Article Type	1 = Outcome evaluation; 2 = Process evaluation; 3 = Protocol (e.g. full protocol for design of RCT); 4 = Intervention description / development paper (i.e. description or development of intervention, not full protocol for study)
Target Behaviour	Add target behaviour(s) as described by author(s).<br><br>Note: Ensure what you are coding is **behaviour** and not outcome (e.g. physical activity not weight loss). If >1, list all targeted.
Underpinning theory/model of behaviour mentioned	0 = No underpinning theory mentioned; 1 = Theory mentioned, unclear if underpins intervention; 2 = Theory guided intervention<br><br>Note: Code as 'Theory Guided Intervention' if theory is explicitly mentioned as underpinning part or all of the intervention.
Intervention Theory	Note: Only code if theory guided intervention (i.e. '2' in previous column)
Intervention Development Framework	0 = None or Unclear<br>1 = Intervention Mapping<br>2 = Precede Proceed<br>3 = BCW<br>4 = MRC framework<br><br>Note: Add to this as needed. Only code as ≥1 if clear that intervention framework guided design (e.g. 'the intervention was developed using the PRECEDE model').
Coded by BCTs (using a taxonomy) in paper	0 = No<br>1 = Partly<br>2 = Yes<br><br>Note: Code as 2 if paper explicitly uses a taxonomy (e.g. v1, CALO-RE) to code intervention content. Citing taxonomy paper is not enough.<br>Code as 1 if some BCTs are mentioned but not all intervention content is coded.

APPENDIX B
Sample Extracted Data for Source Table

SOURCE ID	AUTHOR (YEAR)	YEAR	ARTICLE TYPE	TARGET BEHAVIOUR	THEORY MENTIONED	INT. THEORY	INT. DEV FRAMEWORK	BCT CODED (IN PAPER)
STAN15	Staunton	2015	1	Dental flossing	2	1, 5	0	2
JEMM15	Jemmot	2015	2	Physical Activity	2	2	0	1
TILL99	Tilley	1999	1	Diet	2	1, 3	0	0
SASS14	Sassen	2014	4	Physical Activity	0	N/A	0	0
AUSE02	Ausems	2002	1	Smoking	2	1	0	0

APPENDIX C:
Data Extraction for Links Table

Field	Data
Source ID	Code denoting Source ID (ensure this matches Source ID from Source Table)<br><br>Note: insert a new row for every new BCT-mechanism link; do not add multiple rows for the same link (i.e. insert only one row even if the link appears multiple times).
BCT	BCT number (01-93)<br><br>See Appendix E for codes
BCT confidence	1 = One plus (present in all probability)<br>2 = Two plus (present beyond all reasonable doubt)
Mechanism of Action	Mechanism of Action (take wording directly from paper)<br><br>Note: If a BCT is not linked to any mechanisms, do not add to link spreadsheet – add only to BCT coding mastersheet.
Definition	Copy and paste definition of mechanism as described by authors<br><br>If no definition given:<br>- If mechanism(s) measured, include items used to measure<br>- If not defined, write 'not defined'
Target Behaviour	Insert Target Behaviour of individual BCT (e.g. condom purchasing)
Explicit	1 = BCT hypothesised to change behaviour through mechanism but some inference needed<br>2 = BCT explicitly hypothesised to change behaviour through mechanism<br><br>Note: Code 1 if indirect link (e.g. BCT -> intention through attitudes)
Grouped	1 = 1 BCT linked to 1 MoA<br>2 = Group of BCTs explicitly linked to one MoA or one BCT linked to group of MoAs
Empirically Tested	0 = No<br>1 = Mechanism measured<br>2 = BCT-mechanism link tested<br><br>Note: For each link, code 1 when the impact of the intervention on the mechanism was measured (e.g. pre- to post-intervention) and code 2 when the impact of the individual BCT on the mechanism was tested. If protocol/description paper, code N/A
Page	Insert page number from source where link is mentioned
Location	1 = Introduction<br>2 = Method<br>3 = Results<br>4 = Discussion<br>5 = Supplementary File<br>6 = Link from another (earlier) paper (e.g. protocol

Comment	Where relevant, include short comment with text from report relating to link

APPENDIX D
Sample Extracted Links Table

ID	BCT	BCT Confidence	MECHANISM	DEFINITION	TARGET BEHAVIOUR	EXPLICITNESS	GROUPED	EMPIRICALLY TESTED
STAN15	21	2	Perceived control	The extent to which individuals feel they have control over behaviours	Flossing Behaviour	2	2	1
STAN15	25	2	Perceived control	The extent to which individuals feel they have control over behaviours	Flossing Behaviour	2	2	1
STAN15	25	2	Intrinsic Motivation	Engaging in a behaviour spontaneously and for inherent interest and enjoyment (i.e. self-determined)	Flossing Behaviour	2	2	1
STAN15	48	2	Extrinsic Motivation	Engaging in a behaviour to please others	Flossing Behaviour	2	2	1
JEMM15	17	1	Subjective Norm	Not defined but measured by: ‘Most people who are important to me would think…’	Physical Activity	2	1	1
JEMM15	20	1	Self-efficacy	Not defined (= ‘perceived behavioural control’) but measured by: ‘I am confident that I can overcome obstacles that might prevent me from …’	Physical Activity	2	2	1

APPENDIX E
BCT CODES FOR LINK EXTRACTION

CODE	BCT NAME
**1**	Goal setting (behaviour)
**2**	Problem solving
**3**	Goal setting (outcome)
**4**	Action planning
**5**	Review behaviour goals
**6**	Discrepancy between current behaviour and goal
**7**	Review outcome goals
**8**	Behavioural contract
**9**	Commitment
**10**	Monitoring of behaviour by others without feedback
**11**	Feedback on behaviour
**12**	Self-monitoring of behaviour
**13**	Self-monitoring of outcomes of behaviour
**14**	Monitoring of outcomes of behaviour without feedback
**15**	Biofeedback
**16**	Feedback on outcomes of behaviour
**17**	Social support (unspecified)
**18**	Social support (practical)
**19**	Social support (emotional)
**20**	Instruction on how to perform the behaviour
**21**	Information about Antecedents
**22**	Re-attribution
**23**	Behavioural experiments
**24**	Information about health consequences
**25**	Salience of consequences
**26**	Information about social and environmental consequences
**27**	Monitoring of emotional consequences
**28**	Anticipated regret
**29**	Information about emotional consequences
**30**	Demonstration of the behaviour
**31**	Social comparison
**32**	Information about others' approval
**33**	Prompts/cues
**34**	Cue signalling reward
**35**	Reduce prompts/cues
**36**	Remove access to the reward
**37**	Remove aversive stimulus
**38**	Satiation
**39**	Exposure
**40**	Associative learning
**41**	Behavioural practice/rehearsal

**42**	Behaviour substitution
**43**	Habit formation
**44**	Habit reversal
**45**	Overcorrection
**46**	Generalisation of the target behaviour
**47**	Graded tasks
**48**	Credible source
**49**	Pros and cons
**50**	Comparative imagining of future outcomes
**51**	Material incentive (behaviour)
**52**	Material reward (behaviour)
**53**	Non-specific reward
**54**	Social reward
**55**	Social incentive
**56**	Non-specific incentive
**57**	Self-incentive
**58**	Incentive (outcome)
**59**	Self-reward
**60**	Reward (outcome)
**61**	Future punishment
**62**	Pharmacological support
**63**	Reduce negative emotions
**64**	Conserving mental resources
**65**	Paradoxical instructions
**66**	Restructuring the physical environment
**67**	Restructuring the social environment
**68**	Avoidance/reducing exposure to cues for the behaviour
**69**	Distraction
**70**	Adding objects to the environment
**71**	Body changes
**72**	Identification of self as role model
**73**	Framing/reframing
**74**	Incompatible beliefs
**75**	Valued self-identity
**76**	Identity associated with changed behaviour
**77**	Behaviour cost
**78**	Punishment
**79**	Remove reward
**80**	Reward approximation
**81**	Rewarding completion
**82**	Situation-specific reward
**83**	Reward incompatible behaviour
**84**	Reward alternative behaviour
**85**	Reduce reward frequency

**86**	Remove punishment
**87**	Verbal persuasion about capability
**88**	Mental rehearsal of successful performance
**89**	Focus on past success
**90**	Self-talk
**91**	Imaginary punishment
**92**	Imaginary reward
**93**	Vicarious consequences

Appendix F: 26 Mechanisms of Action

Mechanisms of action are defined as "the processes through which a BCT affects behaviour". These 26 mechanisms of action are taken from the 14 theoretical domains as described in the Theoretical Domains Framework (Cane et al., 2012) and the 12 most frequently occurring mechanisms derived from a set of 83 behaviour change theories (Michie, West, et al., 2014):

	**Mechanism Definition**	**Mechanism Label**
1.	An awareness of the existence of something	Knowledge
2.	An ability or proficiency acquired through practice	Skills
3.	A coherent set of behaviours and displayed personal qualities of an individual in a social or work setting	Social/ Professional Role and Identity
4.	Beliefs about one's ability to successfully carry out a behaviour	Beliefs about Capabilities
5.	Confidence that things will happen for the best or that desired goals will be attained	Optimism
6.	Beliefs about the consequences of a behaviour (i.e. perceptions about what will be achieved and/or lost by undertaking a behaviour, as well as the probability that a behaviour will lead to a specific outcome)	Beliefs about Consequences
7.	Processes by which the frequency or probability of a response is increased through a dependent relationship or contingency with a stimulus or circumstance	Reinforcement
8.	A conscious decision to perform a behaviour or a resolve to act in a certain way	Intention
9.	Mental representations of outcomes or end states that an individual wants to achieve	Goals
10.	Ability to retain information, focus on aspects of the environment and choose between two or more alternatives	Memory, Attention and Decision Processes
11.	Aspects of a person's situation or environment that discourage or encourage the behaviour	Environmental Context and Resources
12.	Those interpersonal processes that can cause oneself to change one's thoughts, feelings or behaviours.	Social Influences

13.	A complex reaction pattern involving experiential, behavioural, and physiological elements	Emotion
14.	Behavioural, cognitive and/or emotional skills for managing or changing behaviour	Behavioural Regulation
15.	The attitudes held and behaviours exhibited by other people within a social group	Norms
16.	One's ***perceptions*** of what most other people within a social group believe and do	Subjective Norms
17.	The general evaluations of the behaviour on a scale ranging from negative to positive	Attitude towards the behaviour
18.	Processes relating to the impetus that gives purpose or direction to behaviour and operates at a conscious or unconscious level	Motivation
19.	One's conception and evaluation of oneself, including psychological and physical characteristics, qualities and skills	Self-image
20.	Deficit of something required for survival, well-being or personal fulfilment	Needs
21.	Moral, social or aesthetic principles accepted by an individual or society as a guide to what is good, desirable or important	Values
22.	Processes through which current behaviour is compared against a particular standard	Feedback Processes
23.	A process by which thoughts, feelings and motivational states observed in others are internalised and replicated without the need for conscious awareness	Social Learning / Imitation
24.	Processes by which behaviour is triggered from either the external environment, the performance of another behaviour, or from ideas appearing in consciousness	Behavioural Cueing
25.	Evaluations of an object, person, group, issue or concept on a scale ranging from negative to positive	General Attitudes / Beliefs
26.	Perceptions of the likelihood that one is vulnerable to a threat	Perceived susceptibility/ vulnerability
