# Supplementary material for: Behavior Change Techniques and Their Mechanisms of Action: A Synthesis of Links Described in Published Intervention Literature
Source: Ann Behav Med. 2018 Oct 10;53(8):693–707. doi: 10.1093/abm/kay078 (PMC6636886; doi:10.1093/abm/kay078)
Supplement: kay078_suppl_Supplementary_File_3 [file kay078_suppl_supplementary_file_3.pdf]

Supplementary File 3: Guidelines for Categorisation of Mechanisms

- Read and re-read the 26 MoA labels and definitions (in BCT-MoA Link Coding Guidelines)
- Ensure all decisions are based on **definitions**, not labels
- If definition is given and clearly fits **one** of the 26 MoA definitions, add label and number of MoA in spreadsheet
- If definition is given and fits into **more than one** of the 26 MoA definitions (e.g. a definition encompasses both attitude towards behaviour AND beliefs about consequences), highlight in yellow for review
- If definition is given and unsure which of the 26 MoA definitions it fits into, highlight in yellow for review
- If **no** definition given:
    - Double-check PDF and add any relevant information where possible (e.g. how MoA was measured or what theory it came from)
        - If no additional information found and/or still unclear, highlight in yellow for review
    - If the definition says ‘as per [theory]’ – check the theory definition, if not clear, highlight in yellow for review
- Where categorisation of MoAs **within one paper** means that there are multiple identical links, highlight these in yellow. For example, where intrinsic motivation is linked to BCT X and Y, and extrinsic motivation is linked to BCT X and Y, and these are both categorised into ‘Motivation’ (meaning Motivation is linked to BCT X and Y twice), highlight these BCT numbers in yellow in the Links spreadsheet (see examples of this in rows 51-58).
- Where no definition given but label is exactly label of one of the 26 MoAs, ok to categorise as such (this includes self-efficacy → beliefs about capabilities, outcome expectancies → beliefs about consequences, and social support → social influences).
- For uncertain MoAs:
    - Check whether construct is similar/identical to one of the component constructs of the TDF domain or frequently occurring MoA
    - If no definition or information given about construct in paper, and label is not similar to any MoA, code as ‘other’.

Examples of Construct Definitions from Theories (to be used where authors define an MoA ‘as per X theory’)

**Construct Label**	**Construct Definition (from theory)**	**Mechanism of Action Categorisation**
Intention	TPB: The cognitive representation of one's readiness to perform the behaviour; considered to be the immediate antecedent of behaviour	Intention (8)
Attitude	TPB: Favourable or unfavourable evaluation or appraisal of the behaviour	Attitude towards the behaviour (17)
Perceived Susceptibility	HBM: The extent to which one believes one is at risk of contracting a particular disease or developing a particular health problem	Perceived Susceptibility/ Vulnerability (26)
Perceived Threat	HBM: Perceptions of the threat posed by a disease or health problem, based on one's perceived susceptibility to, and the perceived severity of, a disease or health	Perceived Susceptibility/ Vulnerability (26)

	problem	
Perceived Behavioural Control	TPB: The perceived ability to perform the behaviour	Beliefs about Capabilities (4)
Self-Efficacy	SCT: Perceived judgements of one's ability to cope effectively in different circumstances	Beliefs about Capabilities (4)
Self-Efficacy	SET: Belief that one is capable of carrying out a specific behaviour that will lead to desired outcomes	Beliefs about Capabilities (4)
Behavioural Intention	HAPA: A conscious decision to perform a behaviour	Intention (8)
Self-Regulation	HAPA (Action Control?): Actual or perceived environmental factors that prevent performance of an intended behaviour	Behavioural Regulation (14)
Motivation	I-change: One's intention to carry out a particular type of behaviour; determined by attitudes, social influences and self-efficacy	Intention (8)

Inter-Coder Reliability

**Index**	**Reliability (of MoAs not highlighted in yellow)**	**# MoAs categorised before reliability achieved**
**Percentage Agreement**	90%	20
